# Supplementary material for: A role for the periplasmic adaptor protein AcrA in vetting substrate access to the RND efflux transporter AcrB
Source: Sci Rep. 2022 Mar 19;12:4752. doi: 10.1038/s41598-022-08903-9 (PMC8934357; doi:10.1038/s41598-022-08903-9)
Supplement: Supplementary file 1 — Supplementary Information. [file 41598_2022_8903_MOESM1_ESM.pdf]

Supplementary Material

A role for the periplasmic adaptor protein AcrA in vetting substrate access to the RND efflux transporter AcrB

Ilyas Alav, Vassiliy N. Bavro, Jessica M. A. Blair

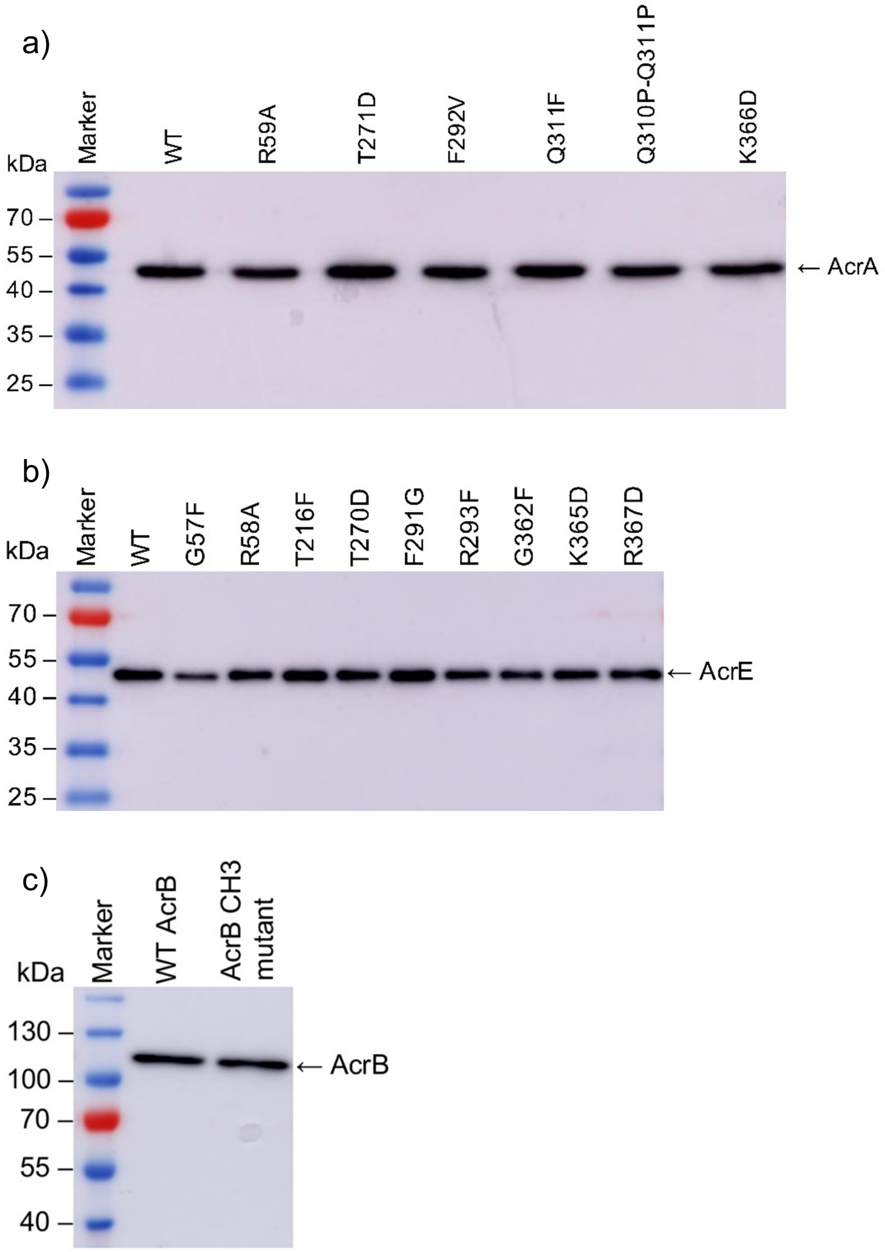

Figure S1. Western blotting of mutant proteins. a) Wild type and mutant AcrA were in expressed in the *Salmonella* Typhimurium SL1344 Δ4PAP strain from p*acrA* plasmids. **b)** Wild type and mutant AcrE were in expressed in the *Salmonella* Typhimurium SL1344 Δ4PAP Δ*acrF* strain from p*acrE* plasmids. **c)** Wild type and the AcrB channel 3 (CH3) mutant (A33W T37W N298W) were in expressed in the *Salmonella* Typhimurium SL1344 Δ4PAP Δ*acrB* strain from p*acrAB* plasmids. Membrane fractions were harvested, separated on a 12% SDS-PAGE gel for AcrA and AcrE or 8% SDS-PAGE gel for AcrB, and transferred to a PVDF membrane. The His-tagged proteins were blotted using anti 6x-His tag HRP-conjugated monoclonal antibody and detected using ECL substrate. PageRuler Prestained Protein Ladder (Thermo Scientific, USA) was used as a molecular weight marker.

Table S1. List of primers used for site-directed mutagenesis reactions.

Primer name	Primer sequence
AcrA_R59A_F	CTTCCGGGTGCTACCGTTGCTTACCGTATC
AcrA_R59A_R	GCAACGGTAGCACCCGGAAGTTCAGTTGTG
AcrA_T270A_F	GTTGACCAAGCCACCGGGTCTATTACTTTG
AcrA_T270A_R	CCCGGTGGCTTGGTCAACGGTCACGTCGG
AcrA_T270D_F	GACCGTTGACCAAGACACCGGGTCTATTAC
AcrA_T270D_R	GACCCGGTGTCTTGGTCAACGGTCACGTCG
AcrA_T271A_F	GTTGACCAAACCGCCGGGTCTATTACTTTGC
AcrA_T271A_R	GTAATAGACCCGGCGGTTTGGTCAACGGTC
AcrA_T271D_F	GTTGACCAAACCGACGGGTCTATTACTTTGC
AcrA_T271D_R	GTAATAGACCCGTCGGTTTGGTCAACGGTC
AcrA_G272A_S273A_F	CAAACCACCGCGGCTATTACTTTGCGCGCC
AcrA_G272A_S273A_R	GTAATAGCCGCGGTGGTTTGGTCAACGGTCAC
AcrA_F292V_F	CCAGGAATGGTCGTTCGCGCACGTCTGC
AcrA_F292V_R	CGAACGACCATTCCTGGCAATAAGGTGTG
AcrA_Q310P_Q311P_F	CTGGTTCCACCACCGGGCGTTACCCGTACTC
AcrA_Q310P_Q311P_R	GTAACGCCCGGTGGTGGAACCAGTAATGCCG
AcrA_Q311F_F	GTTCCACAATTCGGCGTTACCCGTACTCC
AcrA_Q311F_R	GTAACGCCGAATTGTGGAACCAGTAATGC
AcrA_P317G_F	GTTACCCGTACTGGACGCGGCGATGCCAC
AcrA_P317G_R	TCGCCGCGTCCAGTACGGGTAACGCCCT
AcrA_P317F_F	GTTACCCGTACTTTCCGCGGCGATGCCACG
AcrA_P317F_R	CGCCGCGGAAAGTACGGGTAACGCCCTGTTG
AcrA_R318F_F	CGTACTCCATTCGGCGATGCCACGGTGCTG
AcrA_R318F_R	CATCGCCGAATGGAGTACGGGTAACGCCCTG
AcrA_I343F_G344F_F	GCCAGGCGTTCTTCGATAAGTGGCTGGTGAC
AcrA_I343F_G344F_R	CACTTATCGAAGAACGCCTGGCTTGCGACG
AcrA_Q365F_F	GCGGGCTGTTCAAAGTACGTCCTGGCGCAC
AcrA_Q365F_R	GACGTACTTTGAACAGCCCGCTGACGACTAC
AcrA_K346A_F	AGCCAGGCGATCGGCGATGCGTGGCTGGTG
AcrA_K346A_R	CACCAGCCACGCATCGCCGATCGCCTGGCT
AcrA_K366D_F	GGCTGCAAGATGTACGTCCTGGCGCACAGG
AcrA_K366D_R	GGACGTACATCTTGCAGCCCGCTGACGAC
AcrA_R368A_F	CAGCGGGCTGCAAAAAGTAGCTCCTGGCGCA
AcrA_R368A_R	TGCGCCAGGAGCTACTTTTTGCAGCCCGCTG
AcrA_R368F_F	GCAAAAAGTATTTCCTGGCGCACAGGTTAAAG
AcrA_R368F_R	CGCCAGGAAATACTTTTTGCAGCCCGCTGAC
AcrE_G57F_F	GTAACGACCGAACTTCCCTTCCGTACGTCCGCATTTCGC
AcrE_G57F_R	GCGAAATGCGGACGTACGGAAGGGAAGTTCGGTCGTTAC

AcrE_R58A_F	ACCGAACTTCCCGGAGCTACGTCCGCATTTCG
AcrE_R58A_R	CGAAATGCGGACGTAGCTCCGGGAAGTTCGGT
AcrE_T216F_F	CGATCCGATTTATGTCGACGTGTTCCAATCAAGCAACGACTTTATGC
AcrE_T216F_F	GCATAAAGTCGTTGCTTGATTGGAACACGTCGACATAAATCGGATCG
AcrE_T270D_F	GTTACCGTAGATGAAAGCGACGGCTCTATCACGCTCAG
AcrE_T270D_R	CTGAGCGTGATAGAGCCGTCGCTTTCATCTACGGTAAC
AcrE_F291G_F	CTGCTTCCCGGTATGGGTGTTCGCGCCCGCAT
AcrE_F291G_R	ATGCGGGCGCGAACACCCATACCGGGAAGCAG
AcrE_R293F_F	GTCTGCTTCCCGGTATGTTTGTTTTCGCCCGCATTGA
AcrE_R293F_R	TCAATGCGGGCGAAAACAAACATACCGGGAAGCAGAC
AcrE_G362F_F	CGATAAGGTCATCGTCAGCTTCTTACAAAAAGCGCGACCG
AcrE_G362F_R	CGGTCGCGCTTTTTGTAAGAAGCTGACGATGACCTTATCG
AcrE_K365D_F	CATCGTCAGCGGCTTACAAGATGCGCGACCGG
AcrE_K365D_R	CCGGTCGCGCATCTTGTAAGCCGCTGACGATG
AcrE_R367D_F	CGGCTTACAAAAAGCGGATCCGGGCGTCCAGGTG
AcrE_R367D_R	CACCTGGACGCCCGGATCCGCTTTTTGTAAGCCG
AcrB_A33W_F	GCGATCCTCAAATTGCCGGTATGGCAATATCCGACGAT
AcrB_A33W_R	ATCGTCGGATATTGCCATACCGGCAATTTGAGGATCGC
AcrB_T37W_F	GGTATGGCAATATCCGTGGATTGCGCCACCAGCA
AcrB_T37W_R	TGCTGGTGGCGCAATCCACGGATATTGCCATACC
AcrB_N298W_F	TGGCTACCGGCGCCTGGGCGCTGGATACCGC
AcrB_N298W_R	GCGGTATCCAGCGCCCAGGCGCCGGTAGCCA

Table S2. Antimicrobial susceptibility of the *Salmonella* Typhimurium SL1344 Δ4PAP strain complemented with mutated versions of AcrA.

		**MIC (µg mL^{-1})**									
**Strain**	**Box no.**	ACR	CLI	CV	DOX	EtBr	ERY	FA	MB	NOV	R6G
WT		256	256	64	512	>1024	128	1024	>1024	512	>1024
Δ4PAP		16	2	2	2	16	2	4	8	1	8
WT complement		<u>64</u>	<u>128</u>	<u>16</u>	<u>64</u>	<u>128</u>	<u>64</u>	<u>256</u>	<u>128</u>	<u>128</u>	<u>128</u>
R59A	**1**	**16**	**2**	**2**	**2**	**32**	**2**	**4**	**16**	**2**	**16**
T270A	4	64	128	16	64	128	64	256	128	128	128
T270D	4	64	128	16	64	128	64	256	128	128	128
T271A	4	64	16	8	32	64	**8**	**64**	**32**	**8**	**32**
T271D	**4**	**16**	**2**	**2**	**2**	**16**	**2**	**4**	**8**	**1**	**8**
G272A-S273A	4	64	128	16	64	128	64	256	128	128	128
F292V	**5**	**16**	**2**	**2**	**2**	**16**	**2**	**4**	**8**	**1**	**8**
Q310P-Q311P	**pre-6**	**16**	**2**	**2**	**2**	**16**	**2**	**4**	**8**	**1**	**8**
Q311F	pre-6	64	128	16	64	64	64	128	128	64	128
P317G	6	64	128	16	64	64	64	128	128	128	128
P317F	6	64	128	16	64	128	64	256	128	128	128
R318F	6	64	128	16	64	128	64	256	128	128	128
I343F-G344F	8	64	128	16	64	128	64	256	128	128	128
K346A	8	64	128	16	64	128	64	256	128	128	128
Q365F	9	64	128	16	64	128	64	256	128	128	64
K366D	9	64	**16**	16	**16**	64	**16**	**16**	128	**16**	64
R368F	9	64	128	16	64	128	64	256	128	128	128

Underlined values highlight values for the Δ4PAP strain complemented with wild type AcrA (WT complement). Bold values are at least two-fold or more different than the parent strain. ACR, acriflavine; CLI, clindamycin; CV, crystal violet; DOX, doxorubicin; EtBr, ethidium bromide; ERY, erythromycin; FA, fusidic acid; MB, methylene blue; NOV, novobiocin; R6G, rhodamine 6G. Box no. indicates the mapping of the mutation to its binding box.

Table S3. Antimicrobial susceptibility of the *Salmonella* Typhimurium SL1344 Δ4PAP Δ*acrF* strain complemented with mutated versions of AcrE.

		MIC (µg mL^{-1})												
**Strain**	**Box no.**	ACR	BZK	CHL	CLI	CV	DOX	EtBr	ERY	FA	MB	MIN	NOV	R6G
WT		256	64	4	512	64	1024	>1024	64	1024	1024	1	512	>1024
Δ4PAP Δ*acrF*		16	4	0.5	1	2	2	16	4	4	8	0.25	1	8
WT complement		256	64	4	128	32	512	>1024	64	512	1024	1	512	>1024
G57F	**1**	**16**	**4**	**0.5**	**4**	**1**	**2**	**16**	**4**	**4**	**8**	**0.25**	**2**	**8**
R58A	**1**	**16**	**4**	**0.5**	**4**	**1**	**2**	**16**	**4**	**4**	**8**	**0.25**	**2**	**8**
T216F	2	256	64	4	128	32	512	1024	64	1024	1024	1	512	1024
T270D	**4**	**16**	**4**	**0.5**	**4**	**1**	**2**	**16**	**4**	**8**	**8**	**0.25**	**1**	**8**
F291G	**5**	**16**	**4**	**0.5**	**4**	**2**	**2**	**16**	**4**	**8**	**8**	**0.25**	**1**	**8**
R293F	**5**	**16**	**8**	**0.5**	**8**	**2**	**8**	**64**	**4**	**32**	**64**	**0.25**	**16**	**64**
G362F	**9**	**32**	**8**	**0.5**	**16**	**2**	**8**	**64**	**4**	**32**	**128**	**0.25**	**16**	**64**
K365D	9	128	64	4	128	16	512	1024	64	256	1024	1	256	1024
R367D	9	256	64	4	128	32	512	1024	64	512	1024	1	256	1024

Underlined values highlight values for the Δ4PAP Δ*acrF* strain complemented with wild type AcrE (WT complement). Bold values are at least two-fold or more different than the parent strain. ACR, acriflavine; BZK, benzalkonium chloride; CHL, chloramphenicol; CLI, clindamycin; CV, crystal violet; DOX, doxorubicin; EtBr, ethidium bromide; ERY, erythromycin; FA, fusidic acid; MB, methylene blue; MIN, minocycline; NOV, novobiocin; R6G, rhodamine 6G. Box no. indicates the mapping of the mutation to its binding box.

Table S4. Antimicrobial susceptibility of the *Salmonella* Typhimurium SL1344 Δ4PAP Δ*acrB* strain complemented with K366D AcrA and the AcrB channel 3 mutation.

	**MIC (µg mL^{-1})**									
	HMMD				PAC					
**Strain**	ERY	DOX	FA	NOV	ACR	BZK	CV	EtBr	MB	R6G
WT	128	1024	1024	512	256	64	64	>1024	1024	>1024
Δ4PAP Δ*acrB*	4	2	4	1	8	2	2	8	8	8
WT AcrAB complement	<u>64</u>	<u>64</u>	<u>128</u>	<u>64</u>	<u>128</u>	<u>32</u>	<u>16</u>	<u>256</u>	<u>256</u>	<u>128</u>
WT AcrA + AcrB CH3 mutation	16	32	32	16	32	8	8	32	32	32
K366D AcrA + WT AcrB	16	16	16	16	128	32	16	128	128	64
K366D AcrA + AcrB CH3 mutation	**4**	**2**	**4**	**1**	**8**	**2**	**2**	**8**	**8**	**8**

Underlined values highlight values for the Δ4PAP Δ*acrB* strain complemented with WT AcrAB. Bold values highlight the MIC values of the Δ4PAP Δ*acrB* strain complemented with K366D AcrA and the AcrB CH3 (A33W T37W N298W AcrB) mutation compared to its single mutation parent strains. HMMD, high-molecular-mass drugs; ERY, erythromycin; DOX, doxorubicin; FA, fusidic acid; NOV, novobiocin; PAC, planar aromatic cation; ACR, acriflavine; BZK, benzalkonium chloride; CV, crystal violet; EtBr, ethidium bromide; MB, methylene blue; R6G, rhodamine 6G.

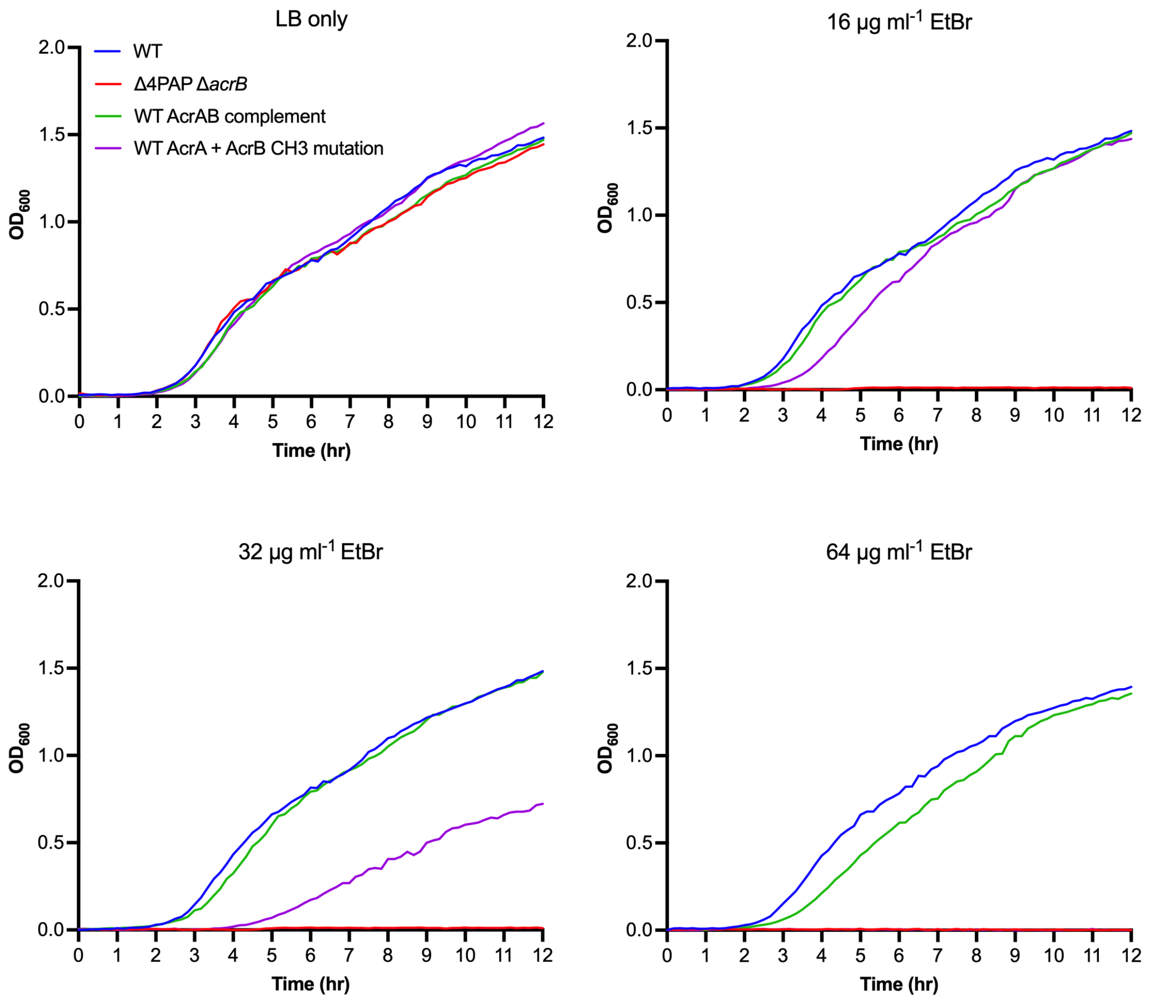

Figure S2. Growth kinetics of the *Salmonella* Typhimurium SL1344 Δ4PAP Δ*acrB* strain complemented with wild type AcrA and the AcrB channel 3 (CH3) mutation in various concentrations of ethidium bromide (EtBr). Data shown are the mean OD_{600} values of three biological replicates. The AcrB CH3 mutation refers to A33W T37W N298W AcrB.

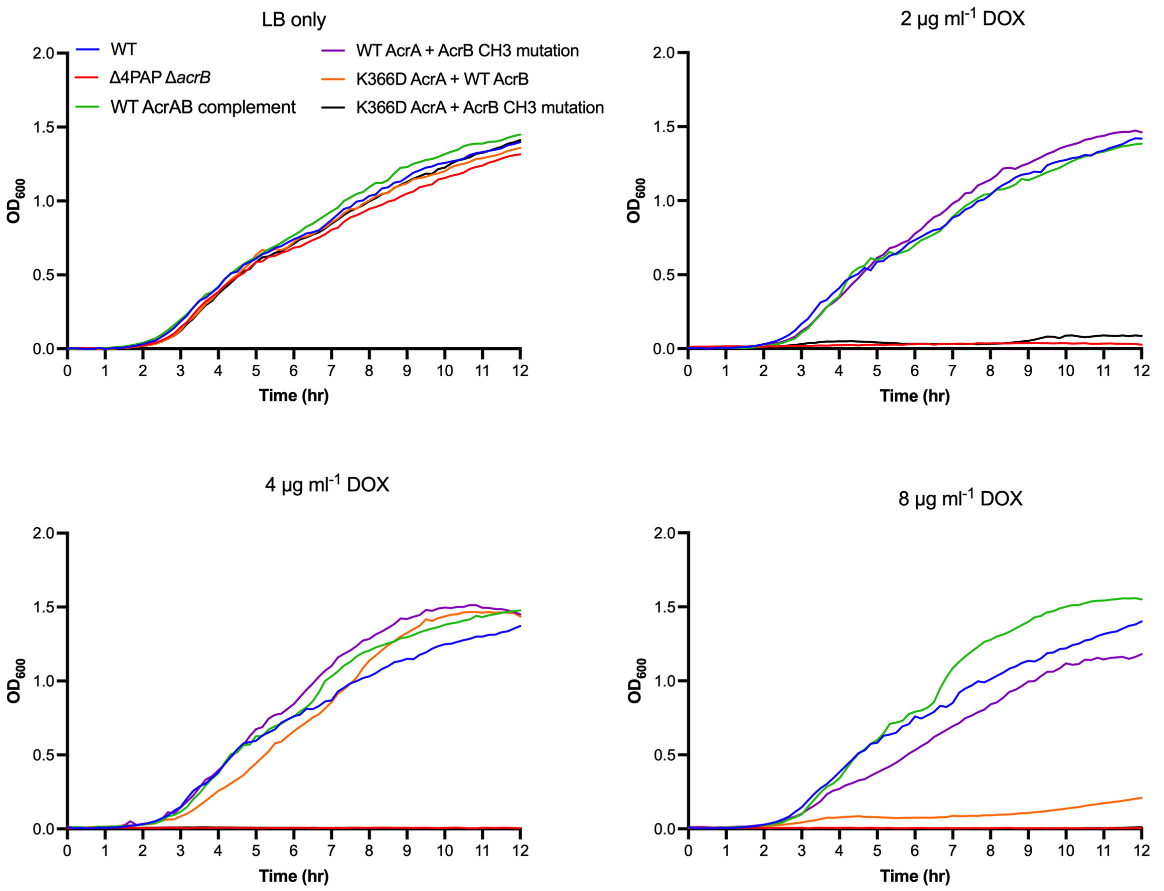

Figure S3. Growth kinetics of the *Salmonella* Typhimurium SL1344 Δ4PAP Δ*acrB* strain complemented with K366D AcrA and the AcrB channel 3 (CH3) mutation in various concentrations of doxorubicin (DOX). Data shown are the mean OD_{600} values of three biological replicates. The AcrB CH3 mutation refers to A33W T37W N298W AcrB.

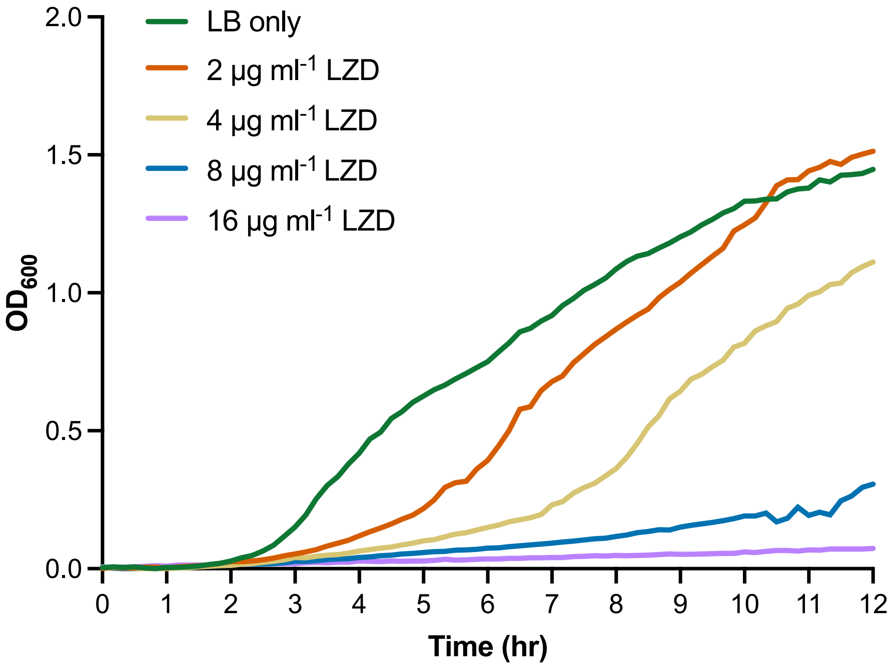

Figure S4. Growth kinetics of the *Salmonella* Typhimurium SL1344 Δ4PAP strain complemented with the K366D AcrA mutation in various concentrations of linezolid (LZD). Data shown are the mean OD_{600} values of three biological replicates.
